# Risk Maps of Lassa Fever in West Africa

**DOI:** 10.1371/journal.pntd.0000388

**Published:** 2009-03-03

**Authors:** Elisabeth Fichet-Calvet, David John Rogers

**Affiliations:** 1 Evolutionary Ecology Group, University of Antwerp, Antwerp, Belgium; 2 Spatial Ecology and Epidemiology Group, Department of Zoology, University of Oxford, Oxford, United Kingdom; University of Texas Medical Branch, United States of America

## Abstract

**Background:**

Lassa fever is caused by a viral haemorrhagic arenavirus that affects two to three million people in West Africa, causing a mortality of between 5,000 and 10,000 each year. The natural reservoir of Lassa virus is the multi-mammate rat *Mastomys natalensis*, which lives in houses and surrounding fields. With the aim of gaining more information to control this disease, we here carry out a spatial analysis of Lassa fever data from human cases and infected rodent hosts covering the period 1965–2007. Information on contemporary environmental conditions (temperature, rainfall, vegetation) was derived from NASA Terra MODIS satellite sensor data and other sources and for elevation from the GTOPO30 surface for the region from Senegal to the Congo. All multi-temporal data were analysed using temporal Fourier techniques to generate images of means, amplitudes and phases which were used as the predictor variables in the models. In addition, meteorological rainfall data collected between 1951 and 1989 were used to generate a synoptic rainfall surface for the same region.

**Methodology/Principal Findings:**

Three different analyses (models) are presented, one superimposing Lassa fever outbreaks on the mean rainfall surface (Model 1) and the other two using non-linear discriminant analytical techniques. Model 2 selected variables in a step-wise inclusive fashion, and Model 3 used an information-theoretic approach in which many different random combinations of 10 variables were fitted to the Lassa fever data. Three combinations of absence∶presence clusters were used in each of Models 2 and 3, the 2 absence∶1 presence cluster combination giving what appeared to be the best result. Model 1 showed that the recorded outbreaks of Lassa fever in human populations occurred in zones receiving between 1,500 and 3,000 mm rainfall annually. Rainfall, and to a much lesser extent temperature variables, were most strongly selected in both Models 2 and 3, and neither vegetation nor altitude seemed particularly important. Both Models 2 and 3 produced mean kappa values in excess of 0.91 (Model 2) or 0.86 (Model 3), making them ‘Excellent’.

**Conclusion/Significance:**

The Lassa fever areas predicted by the models cover approximately 80% of each of Sierra Leone and Liberia, 50% of Guinea, 40% of Nigeria, 30% of each of Côte d'Ivoire, Togo and Benin, and 10% of Ghana.

## Author Summary

Previous studies on the eco-epidemiology of Lassa fever in Guinea, West Africa, have shown that the reservoir is two to three times more infected by Lassa virus in the rainy season than in the dry season. None of the intrinsic variables of the murine population, such as abundance or reproduction, was able to explain this seasonal variation in prevalence. We therefore here investigate the importance of extrinsic environmental variables, partly influenced by the idea that in the case of nephropathia epidemica in Europe contamination of the environment, and therefore survival of the pathogen outside the host, appears to be an important factor in this disease's epidemiology. We therefore made an extensive review of the literature, gathering information about the geographical location of sites where Lassa fever has been certainly identified. Environmental data for these sites (rainfall, temperature, vegetation and altitude) were gathered from a variety of sources, both satellites and ground-based meteorological stations. Several statistical treatments were applied to produce Lassa ‘risk maps’. These maps all indicate a strong influence of rainfall, and a lesser influence of temperature in defining high risk areas. The area of greatest risk is located between Guinea and Cameroon.

## Introduction

Lassa fever (LF) is a viral haemorrhagic fever the pathogenic agent of which is an arenavirus Lassa virus (LASV) first discovered in 1969 in Nigeria, in a missionary nurse living in Lassa, a village close to the border with Cameroon [1]. Lassa fever is widespread in West Africa, affecting 2 million persons per annum with 5,000–10,000 fatalities annually [2]. Since its initial discovery, nosocomial outbreaks of Lassa fever have occurred repeatedly in Sierra Leone: Panguma, Kenema, 1971–83, 1997, Liberia: Zorzor, 1972; Phebe 1972, 1977, 1982; Ganta 1977, 1982 and Nigeria: Jos, 1970, 1993; Onitsha, 1974; Zonkwa, 1975; Vom, 1975–77, Imo, 1989; Lafia, 1993; and Irrua, 2004 [3],[4],[5],[6],[7],[8],[9]. In Guinea, some acute but isolated cases were recorded in hospitals [10] and a single rural outbreak was recorded on the Sierra Leone border in 1982–83 [11]. Between these two areas, namely in Côte d'Ivoire, Ghana, Togo and Benin, no outbreak has ever been recorded, though isolated cases show evidence of viral circulation in that area [12],[13],[14]. Lassa fever therefore appears to have 2 geographically separate endemic areas: the Mano River region (Guinea, Sierra Leone, Liberia) in the West, and Nigeria in the East.

The reservoir host of this virus is the multimammate rat, *Mastomys natalensis*, which was found infected for the first time in Sierra Leone and in Nigeria in 1972 [15],[16], and recently in Guinea [17]. In Upper Guinea, these commensal rodents aggregate in houses during the dry season, and disperse into the surrounding fields in the rainy season, foraging in cultivated areas before harvesting [18]. Villages where LASV-positive rodents have been trapped are all located in rain forest areas or in the transition zone between forest and savannah, within the 1500 mm rainfall isohyet. Rainfall seems to be an important ecological factor because a recent longitudinal study in rodents demonstrated that LASV infection was two to three times higher in the rainy season than in the dry season [18]. There are no studies to date indicating that the virus can survive better in humid than in dry soil, but evidence points in this direction. For example, the recent discovery of a new arenavirus in *Mus minutoides* (Kodoko virus [19]) and of hantavirus in *Hylomyscus simus* (Sangassou virus) in Guinea [20], were both made in rodents trapped in wet habitats, swamps or along river edges. In the USA, many new hantaviruses discovered within the last 15 years are found in damp or wet places such as arroyos or canyons, i.e. Black Creek canal virus, Blue river virus, El moro Canyon virus, Limestone Canyon virus. In the case of Sin Nombre virus, responsible for hemorrhagic fever with pulmonary syndrome, high risk areas are associated with higher elevation and mesic vegetation whereas low risk areas are associated with lower elevation and xeric vegetation. Soil moisture appears to be a key factor explaining the maintenance of this virus in high risk areas [21],[22]. In Europe, the transmission and persistence of Puumala virus, responsible for nephropathia epidemica, seems possible only if indirect transmission through a contaminated environment is included in a mathematical model. The combination of viral dynamics inside and outside the host, rodent demographic patterns and humid periods seems to explain the geographical distribution of this disease [23]. These advances all indicate the possible importance of rainfall patterns and humidity for Lassa Fever. We present our analysis of LF in West Africa in three steps: a first univariate analysis linking LF with high rainfall areas (Model 1) and the other two, multivariate analyses quantifying associations between LASV presence and a number of environmental parameters, derived from earth-observing satellites, that lead to the production of the first predictive risk maps for Lassa fever. One of these multivariate modelling approaches uses step-wise variable selection procedures (Model 2) whilst the other uses random combinations of predictor variables to identify the individual best predictors of LASV presence and absence (Model 3).

## Materials and Methods

### Model 1

#### Disease Data

Nosocomial outbreaks and prevalences of Lassa fever in humans were derived from the dataset, and were placed on a map of West and Central Africa (see table 1 for the detailed references by country). The null prevalences recorded in Cameroon, CAR, Gabon and Congo were derived from samples taken in towns [24],[25],[26], whereas the low prevalence of 5% recorded in Pool region in Congo came from samples taken in villages [27]. Elsewhere, prevalences appear as a mean, estimated regionally from several villages or from hospital staffs. Data on human infections cover the period 1965 to 2007.

**Table 1:** Positive localities recorded from humans and rodents indicating the presence of Lassa virus in West Africa.

Country	Administrative region	Town/village/hospital	Latitude	Longitude	Year	Reference for humans	Reference for rodents
Benin	Borgou department	Bambéréké hosp.	10.23	2.66	1977	[14]	
Burkina	Comoé province	Banfora	10.63	−4.77	1974	[54]	
Congo	Pool region	Ngamambou	−4.33	14.85	1981	[27]	
Cote d'Ivoire	Beoumi prefecture	Beoumi	7.67	−5.57	1970–74	[54]	
Cote d'Ivoire	Duekoue prefecture	Forêt Classée	6.66	−7.07	2000	[12]	
Cote d'Ivoire	Guiglo prefecture	Guiglo	6.54	−7.48	2000	[12]	
Guinea	Faranah prefecture	Bantou	10.07	−10.58	2003–05		[17],[18]
Guinea	Faranah prefecture	Gbetaya	9.84	−11.03	1990–92, 1996–97, 2003–05	[55]	[17],[18],[56]
Guinea	Faranah prefecture	Kamaraya	9.88	−10.75	1990–92	[55]	
Guinea	Faranah prefecture	Sangoyah	9.72	−10.88	1990–92, 1996–97	[55]	[56]
Guinea	Faranah prefecture	Tanganya	10.00	−10.97	2003–05		[17],[18]
Guinea	Faranah prefecture	Tindo	9.97	−10.70	1990–92	[55]	
Guinea	Gueckedou prefecture	Bawa	8.56	−10.03	1990–92, 1996–97	[55]	[56]
Guinea	Gueckedou prefecture	Denguedou	8.49	−10.44	1993, 2005	[57]	[17]
Guinea	Gueckedou prefecture	Fangamandou	8.50	−10.60	1990–92, 1993, 1996–97	[55],[57]	[56]
Guinea	Gueckedou prefecture	Guedembou	8.76	−9.99	1993	[57]	
Guinea	Gueckedou prefecture	Kassadou	8.91	−10.35	1993	[57]	
Guinea	Gueckedou prefecture	Kpolodou	8.85	−10.34	1993	[57]	
Guinea	Gueckedou prefecture	Nongoa Mbalia	8.70	−10.37	1990–92	[55]	
Guinea	Gueckedou prefecture	Owe Jiba	8.48	−10.44	1990–92, 1996–97	[55]	[56]
Guinea	Gueckedou prefecture	Sassani Toli	8.75	−10.30	1990–92	[55]	
Guinea	Gueckedou prefecture	Tekoulo	8.54	−10.01	1993, 1996–97	[57]	[56]
Guinea	Gueckedou prefecture	Telekolo	8.47	−10.43	1990–92	[55]	
Guinea	Gueckedou prefecture	Temessadou	8.66	−10.31	1993	[57]	
Guinea	Gueckedou prefecture	Tomandou	8.50	−10.30	1993	[57]	
Guinea	Kindia prefecture	Madina Oula	9.88	−12.45	1982–83, 1990–92, 1996–97	[11],[55]	[56]
Guinea	Kissidougou prefecture	Bambaya	9.30	−10.10	1996–99	[10]	
Guinea	Kissidougou prefecture	Banankoro	9.18	−9.30	1996–99	[10]	
Guinea	Kissidougou prefecture	Boue	9.01	−9.95	1996–97		[56]
Guinea	Kissidougou prefecture	Fedou	9.20	−9.90	1996–99	[10]	
Guinea	Kissidougou prefecture	Telekoro	9.18	−10.10	1965, 1967, 1968, 1996–99	[10],[14]	
Guinea	Kissidougou prefecture	Yende Milimou	8.89	−10.17	1996–99	[10]	
Guinea	Lola prefecture	Gbah	7.62	−8.55	1990–92	[55]	
Guinea	Lola prefecture	Gbenemou	7.71	−8.52	1990–92	[55]	
Guinea	Lola prefecture	Thuo	7.58	−8.50	1990–92	[55]	
Guinea	Macenta prefecture	Lorlu	8.56	−10.02	1996–97		[56]
Guinea	Nzérékoré prefecture	Bignamou	7.33	−9.10	1996–99	[10]	
Guinea	Nzérékoré prefecture	Dieke	7.35	−8.95	1996–99	[10]	
Guinea	Nzérékoré prefecture	Koulenin	7.75	−8.82	1996–99	[10]	
Guinea	Siguiri prefecture	Balato	11.57	−9.32	1990–92	[55]	
Guinea	Yomou prefecture	Bamakama	7.72	−9.27	1990–92, 1996–97	[55]	[56]
Guinea	Yomou prefecture	Komore	7.66	−9.26	1990–92	[55]	
Guinea	Yomou prefecture	Waita	7.56	−9.26	1990–92, 1996–97	[55]	[56]
Liberia	Bomi county	Goodrich plantation hosp. ( = Klay)	6.69	−10.87	1980	[58]	
Liberia	Bong county	Suakoko (Phebe hosp.)	7.19	−9.38	1972	[5],[59],[60]	
Liberia	Grand Cape Mont county	Mano river hosp. ( = Kongo)	7.33	−11.14	1980	[58]	
Liberia	Lofa county	Foya Kamara hosp.	8.36	−10.21	1977, 1979, 1980, 1981	[58],[60],[61]	
Liberia	Lofa county	Koindu	8.22	−10.77	1974	[59]	
Liberia	Lofa county	Yielah	7.82	−9.402	1972	[14]	
Liberia	Lofa county	Zigida	8.04	−9.49	1972	[62],[63]	
Liberia	Lofa county	Zorzor hosp.	7.78	−9.43	1969, 1972,1977, 1979, 1980–82	[5],[58],[59],[60]	
Liberia	Nimba county	Ganta hosp.	7.23	−8.98	1982, 2004	[5],[58]	
Liberia	Nimba county	Louplay	6.95	−8.71	2006	[64]	
Liberia	Nimba county	Saglelpie	6.96	−8.84	2007	[65]	
Mali	Segou region	Ntorosso	13.9	5.4	1971	[54]	
Nigeria	Adamawa state	Takum	7.27	9.98	1974	[54]	
Nigeria	Anambra state	Onitsha hosp.	6.17	6.78	1974	[4]	
Nigeria	Benue state	Gboko	7.32	9.00	1987	[66]	
Nigeria	Borno state	Lassa	10.68	13.27	1969	[1]	
Nigeria	Edo state	Ekpoma	6.75	6.13	2001–04	[9]	
Nigeria	Edo state	Ibilo	7.43	6.08	2001–04	[9]	
Nigeria	Edo state	Igarra	7.28	6.10	2001–04	[9]	
Nigeria	Imo state	Aba hosp.	5.12	7.37	1989	[6]	
Nigeria	Imo state	Aboh Mbaise hosp.	5.55	7.20	1989	[6]	
Nigeria	Kaduna state	Rahama	10.42	8.68	1952	[67]	
Nigeria	Nasarawa state	Lafia hosp.	8.48	8.52	1987, 1992–93	[7],[66]	
Nigeria	Ondo state	Ondo	7.10	4.83	1987	[66]	
Nigeria	Plateau state	Bassa	9.93	8.73	1970	[3]	
Nigeria	Plateau state	Fan	8.82	10.90	1977	[14]	
Nigeria	Plateau state	Jos	9.92	8.90	1970, 1972, 1973, 1992–93	[7],[54]	
Nigeria	Plateau state	Ner-Pankshin	9.33	9.45	1972		[16]
Nigeria	Plateau state	Vom	9.73	8.78	1974–75, 1976, 1977	[14]	
Nigeria	Plateau state	Zonkwa	9.78	8.28	1975	[14]	
Nigeria	Sokoto state	Sokoto	13.06	5.25	1971	[54]	
Nigeria	Taraba state	Gongola	8.50	11.50	1987	[66]	
Nigeria	Taraba state	Jalingo	8.88	11.36	2007	[68]	
Sierra Leone	Bo district	Bo hosp.	7.96	−11.74	2001	[69]	
Sierra Leone	Bo district	Gerihun camp	7.93	−11.58	2003	[70]	
Sierra Leone	Bo district	Jimmi camp	7.60	−11.82	2003	[70]	
Sierra Leone	Bombali district (North)	Kamabunyele	9.18	−11.93	1977–1983	[71]	
Sierra Leone	Bombali district (North)	Kathumpe	9.50	−12.23	1977–1983	[71]	[71]
Sierra Leone	Bombali district (North)	Mamaka	9.10	−12.32	1977–1982	[71]	[71]
Sierra Leone	Kailahun district (East)	Daru hosp.	7.99	−10.85	2000	[72]	
Sierra Leone	Kailahun district (East)	Kailahun hosp.	8.28	−10.57	2001	[69]	
Sierra Leone	Kenema district (East)	Bomie/Landoma	8.23	−11.07	1977–83, 1996–97	[71],[73]	
Sierra Leone	Kenema district (East)	Buima	8.27	−11.11	1996–97	[73]	
Sierra Leone	Kenema district (East)	Daabu	7.92	−10.95	1996–97	[73]	
Sierra Leone	Kenema district (East)	Giema	8.20	−11.05	1977–82		[71]
Sierra Leone	Kenema district (East)	Kenema hosp.	7.90	−11.20	1996–97, 1999, 2001–04	[69],[70],[73],[74]	
Sierra Leone	Kenema district (East)	Konia	8.10	−11.02	1977–83	[71]	[71]
Sierra Leone	Kenema district (East)	Kpandebu	8.22	−11.07	1977–83	[71]	[71]
Sierra Leone	Kenema district (East)	Lalehun	8.20	−11.08	1977–82		[71]
Sierra Leone	Kenema district (East)	Largo camp	8.05	−11.12	2003	[70]	
Sierra Leone	Kenema district (East)	Lowoma	8.22	−11.03	1977–82	[71]	[71]
Sierra Leone	Kenema district (East)	Macca	8.15	−11.22	1996–97	[73]	
Sierra Leone	Kenema district (East)	Neama	8.12	−11.00	1977–83	[71]	
Sierra Leone	Kenema district (East)	Niahun	8.00	−11.07	1977–83	[71]	[71]
Sierra Leone	Kenema district (East)	Njakundoma	8.23	−11.05	1977–83	[71]	[71]
Sierra Leone	Kenema district (East)	Nongowa	7.63	−11.40	2003	[70]	
Sierra Leone	Kenema district (East)	Palima/Tongola	8.22	−11.05	1977–83, 1996–97	[71],[73]	[71]
Sierra Leone	Kenema district (East)	Pandebu	8.21	−11.13	1996–97	[73]	
Sierra Leone	Kenema district (East)	Panguma hosp.	8.20	−11.22	1970–75, 1996–97, 2003	[70],[73],[75],[76],[77]	[15]
Sierra Leone	Kenema district (East)	Segbwema hosp.	8.00	−10.95	1975, 1977–83, 1996–97	[73],[77]	[71]
Sierra Leone	Kenema district (East)	Semewabu	8.02	−10.87	1977–83	[71]	
Sierra Leone	Kenema district (East)	Serabu hosp.	7.85	−11.29	1977	[14]	
Sierra Leone	Kenema district (East)	Tokpombu	8.22	−11.09	1996–97	[73]	
Sierra Leone	Kenema district (East)	Tongo field	8.45	−11.12	1972		[15]
Sierra Leone	Kenema district (East)	Tongo hosp.	8.45	−11.28	1970–72, 1996–97	[73],[75]	
Sierra Leone	Kono district	Kono hosp.	8.75	−11.00	2001	[69]	
Sierra Leone	Moyamba district	Taiama camp	8.20	−12.07	2003	[70]	
Sierra Leone	Pujehun district	Pujehun hosp.	7.35	−11.72	2001	[69]	

Year indicates the time of collection.

#### Climatic data

**Figure 1:**
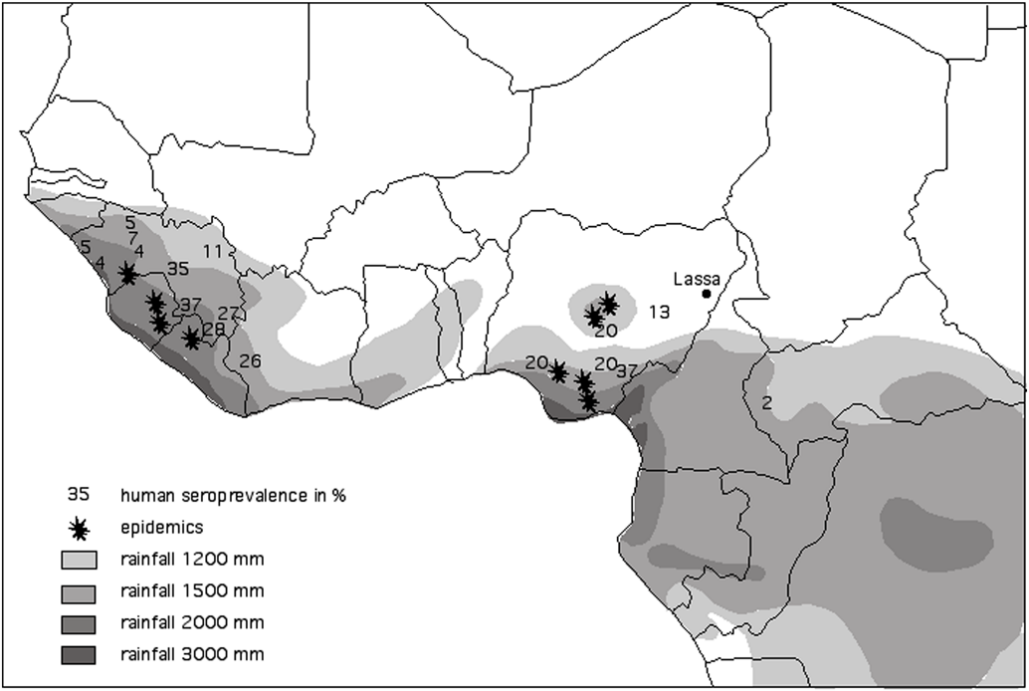
West and Central Africa mean annual rainfall (1951–1989 [28]), Lassa fever nosocomial outbreaks (stars) and human seroprevalence (numbers in %).

A synoptic rainfall map of West Africa was obtained from L'Hôte&Mahé [28] and is shown in Figure 1. This synoptic map is derived from rainfall records for the period 1951 to 1989. In West Africa, the highest rainfall regions are located either side of the Dahomey gap, which separates the 2 great rainforest zones of Guinea and Congo, each region receiving more than 1500 mm of rainfall per year. On the western side, the region includes Guinea, Sierra Leone, Liberia, the extreme West of Côte d'Ivoire and coastal Ghana. The eastern side includes the Congolese zone and south eastern Nigeria (Figure 1).

### Models 2 and 3

#### Disease data

The new Lassa fever database was developed with all indications of Lassa fever presence in West Africa in the period 1965 to 2007. These indications included sero- and virologically positive rodents and human beings. For the rodents, all the localities where *M. natalensis* was screened for LASV were included. Localities were defined as positive when at least one *M. natalensis* was positive, and negative when none was infected. Because of the heterogeneous data for humans, the database was more complicated to establish. The localities were defined as positive when clinical cases were confirmed by a laboratory test or when sampled populations had a seroprevalence ≥10%. The ‘negative’ localities were defined when seroprevalence was <10%. This cut off was defined on the basis of the combined screening of both rodents and humans in the same locality. Rodents were always negative when seroprevalence in humans was <10%. This low human prevalence could be due to the movement of infected humans into an area without infection, whereas one positive rodent always indicates local transmission of LASV. Rodent and human data were acquired from an extensive review of the literature (Table 1).

The latitude and longitude of each recorded locality were then derived from the National Geographic Agency database (http://earth-info.nga.mil/gns/html/namefiles.htm). Because data on rodent infections came mostly or only from targeted samples of these animals, whereas it is assumed that the distribution of human infections is more likely to reflect the distribution of Lassa fever in humans, only data referring to the latter were used in the models presented here. Data referring to humans and rodents were also modelled, but are not presented here because they add only 8 new points, and make little difference to the final map (all data are recorded in Table 1).

#### Environmental variables

Sets of environmental data were derived from remotely sensed imagery from the MODIS instrument on board the NASA Terra satellite for the period 2001–2005 [29] and the processed version 4 of these data were downloaded from NASA's EOS data gateway (http://edcimswww.cr.usgs.gov/pub/imswelcome/). A complete description of the MODIS satellite data used to make these maps, and their processing, is provided by Scharlemann et al 2008 [30]. Data for daytime and night-time land surface temperature (dLST and nLST respectively; MOD11A2 datasets) are available as 8-day composites (compositing removes many of the problems associated with cloud contamination in individual images) [31], whilst data for the Middle Infra Red channel (MODIS band 7, 2105–2155 nm, closest spectrally to the NOAA/AVHRR Channel 3 found useful in previous distribution studies), for the Normalised Difference Vegetation Index (NDVI = [near infrared (NIR)−RED]/[NIR+RED], where NIR is MODIS band 2 and RED is band 1, 841–876 nm and 620–670 nm, respectively) and for the Enhanced Vegetation Index (EVI = (2.5 * [[NIR−RED]/[NIR+6.0 * RED−7.5 * BLUE+1.0]], where BLUE is MODIS band 3, 459–479 nm and NIR and RED are as described above for NDVI) were all derived from 16-day composites after nadir Bidirectional Reflectance Distribution Function (BRDF)-adjustment (MOD43B4 dataset) [32]. The BRDF adjustment removes directional effects of view angle and illumination, providing reflectance values as if every pixel were viewed from nadir, an important correction especially for any channel involving human visible wavelengths. All the MODIS data were available at a nominal resolution of *c*. 1 km at the equator and in the Sinusoidal projection. In addition to thermal and vegetation index data from MODIS a series of monthly rainfall images was obtained from the CMORPH project that uses a variety of satellite data to generate precipitation estimates within the latitudinal range of ±60 degrees [33].

The MODIS and CMORPH data were then temporal Fourier processed to extract, for each channel, a mean, the amplitudes and phases of the annual, bi-annual and tri-annual cycles (i.e. the Fourier harmonics corresponding to these frequencies), the minimum and maximum of the fitted signal and the variance of the original signal. Temporal Fourier processing produces a set of orthogonal (i.e. uncorrelated) variables that capture important elements of habitat seasonality that is often an important driver of vector-borne and other diseases [34],[35]; the particular problems of temporal Fourier processing of MODIS data (and their solutions) are described in Scharlemann et al 2008 [30]. In addition to the satellite variables, the descriptor datasets also included a digital elevation image (DEM) derived from GTOPO30 [36]. All the Fourier variables and the GTOPO30 layer were resampled (by bi-linear interpolation) initially to a resolution of 1/120^th^ degree in the Geographical (latitude/longitude) ‘projection’ and these were then progressively averaged (1/60^th^, 1/30^th^ etc.) to a resolution of 1/15^th^ of a degree, giving a total of 51 Fourier and other (DEM) variables for modelling purposes. All modelling was carried out at this resolution, at which there were 94 unique database records of LASV presence in humans across West Africa. This total number of datapoints is less than the number of human records in Table 1, because some of the records fell within the same pixels at the spatial resolution of the analysis.

#### Statistical techniques

There are many different approaches to mapping species' distributions, recently reviewed by Elith et al [37]. The approach adopted here is described in detail in Rogers 2006 [38] and is based on non-linear maximum likelihood discriminant analysis techniques. For this approach we needed to identify not only areas of presence of each of the cases (from the database), but also equivalent areas of absence. There were insufficient records of absence in the database itself, so an alternative approach was followed, and one thousand points no closer that 0.5 degrees and no farther than 10 degrees away from any of the presence points in the database were chosen at random across West Africa. Because the rodent hosts occur much more extensively across West Africa than does LF, many of these randomly generated absence points fell within the distribution limits of these vertebrate hosts. Thus the models constructed were designed specifically to distinguish the presence and absence of the disease in humans, and not of the hosts of the disease. All satellite and other data were then extracted for both the presence and absence points (hereafter the ‘training set’). These data were first clustered within SPSS for Windows (version 13.0, copyright SPSS Inc., 1989–2004), using the means maxima and minima of each of the MODIS channels, and also the DEM, to produce cluster assignments of the presence and absence data that ran from 1 to 8 clusters each. Within the model the user selected the required combination of numbers of presence and absence clusters at the start of each model run. The LF models described here all used two absence and either one or two presence clusters.

Because of the incomplete nature of the presence (and presumably absence) data in each dataset, it was decided to bootstrap sample the training set data one hundred times, to produce a series of modelled predictions which were averaged to produce the final output map for the disease. Each bootstrap sample contained equal numbers of presence and absence points (this tends to maximise model accuracy; [39]) randomly drawn from the training set, sampled with replacement. The relationship between the bootstrap sample and the training set is imagined to be the same as that between the training set and the entire real world of which the training set itself is a sample. By modelling each bootstrap sample separately, and then averaging the results, it should be possible to establish the variability of model predictions arising from the incomplete sampling of the real world that the training set represents.

#### Model 2 variable selection

Each model involved step-wise inclusive selection of the predictor variables to maximise a goodness of fit criterion; kappa the index of agreement, the area under the curve (AUC) or the Akaike corrected Information Criterion (AICc), all described in Rogers 2006 [38]; a maximum of ten predictor variables was selected for each bootstrap model, but model efficiency (as judged by the AICc) was often highest with fewer than 10 variables; where this applied the final prediction was made using this lower total number of variables. Results for each of the 100 models were kept separate and later brought together to generate accuracy statistics, and to discover whether or not particular variables were consistently included in the predictor datasets. This was done by establishing the mean ranking of each variable in the model selections. The variable selected first in any model run was given a rank of 1, the one selected second a rank of 2, and so on, up to rank 10 for the tenth variable. All non-selected variables in that model run were given a rank of 11. By averaging the ranks of each variable across all models it was possible to establish that variable's importance in the overall predictions.

#### Model 3 variable selection

The problems of step-wise variable selection are well documented; the occurrence of one variable within a dataset can exclude a closely correlated variable that may in fact be more important in determining a disease's distribution. The end product of step-wise selection is therefore a group of variables that are often not strongly correlated with each other, but which are more strongly correlated with those variables left out of the selection. The question then arises about the real importance of the individual variables in determining any particular distribution. Burnham and Anderson [40] suggest a way of answering this important question, and this was followed here. Many random combinations of 10 variables from the entire predictor dataset were made, sampling without replacement (i.e. no variable occurred twice in the same combination), with each variable finally occurring one thousand times across all combinations. Each combination (of 10 variables) was then used to construct a model of LASV distribution using the same bootstrap samples as before. Model accuracy was measured by the corrected Akaike Information Criterion (AICc, a smaller value indicating a better model). Once all the models had been constructed, the mean AICc value of all models containing each variable in turn was calculated, and these mean values were then finally ranked, lowest to highest. The variable giving the lowest mean AICc is then regarded as the ‘best’ predictor of LASV since, regardless of the other (random) variables with which it was associated in the full set of models in which it occurred, those models were overall better than models involving any other single variable. The variable giving the next lowest AICc was the second best individual predictor; and so on.

The difference between the step-wise selected sets of variables (Model 2) and the list of top-ten variables produced by the combination method described above (Model 3) is analogous to the difference between a team (e.g. of footballers) and the top ten runners in an Olympic race. The team players co-operate with each other to win the football match; whilst no individual player may stand out from all the rest, it is the individual's ability to work well with the others that wins the match. In contrast, each runner in a race is competing against all the others. The winner is clearly better than the one who came second who, in turn is better than the one who came third; there is no cooperation between them. They are all collectively better than all the other runners in the race, but this is a result of individual, not collective, ability. It is unlikely that the top ten runners in an Olympic race would make a very good, co-operative team of footballers (and *vice versa*), so the team selection and the individual selection methods explored in Models 2 and 3 are unlikely to come up with the same results. Differences between them may however be illuminating.

In both Models 2 and 3 the selected sets of predictor variables were used within each bootstrap model to generate an image of the posterior probability for each image pixel of belonging to the category of presence pixels as defined within that model. Posterior probabilities are on the scale from 0.0 to 1.0 and a probability in excess of 0.5 is taken as indicating presence. The 100 images from each set of bootstrap samples in each model run were then averaged to produce a single output risk map for the disease.

## Results

### Model 1

Figure 1 shows the location of LF outbreaks (or areas of high human seroprevalence) from 1951 to 1989. The Jos plateau in Nigeria receives more rainfall than the surrounding areas and is disconnected from the wet coastal area by lowland areas of lower rainfall. Only the initial case in Lassa (800 mm/year) is located outside the high rainfall area. The map in Figure 1 suggests that areas with between 1200 mm and 1500 mm of rainfall per year are at relatively low risk of LF; areas with above 1500 mm have a much higher risk and, finally, areas with in excess of 3000 mm of rainfall annually appear to be at zero risk (i.e. had no outbreaks of LF in that period), although these very high rainfall areas are not widespread.

### Model 2

**Figure 2:**
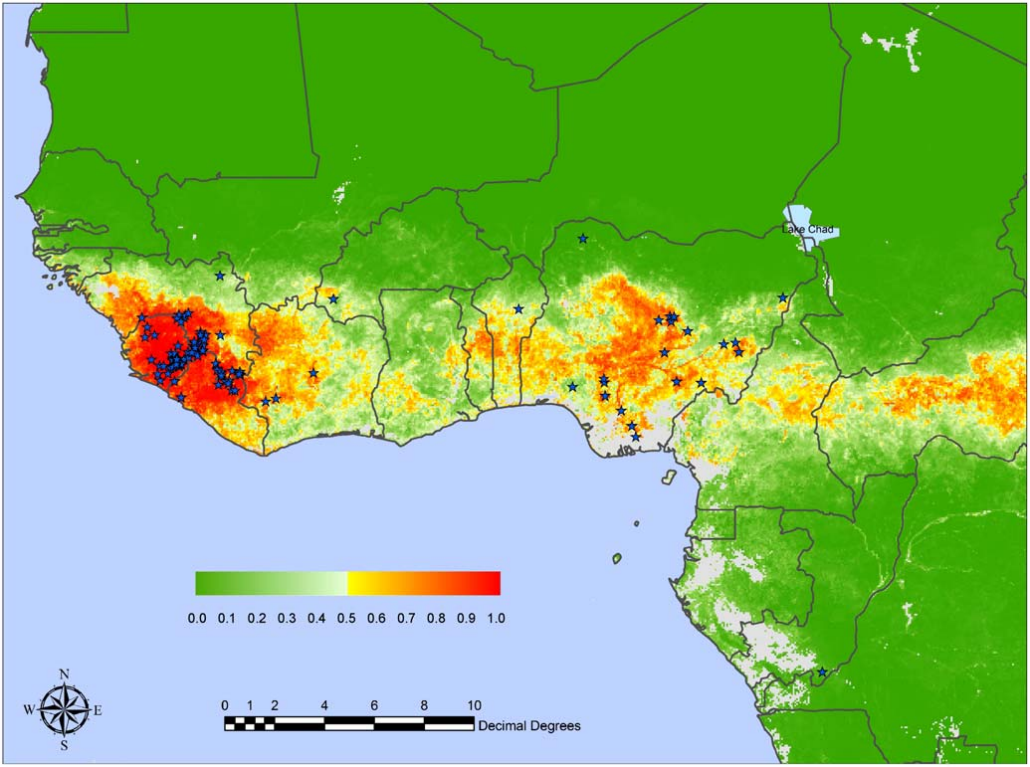
Mean predicted Lassa risk map for West Africa from the Model 2 series with two absence and one presence clusters, with positive localities indicated by stars. The posterior probability colour scale, from 0.0 (no risk) to 1.0 (highest risk) is shown as an inset. Grey areas are either areas with no suitable imagery (because of cloud contamination; coastal Nigeria and Cameroon) or else are so far from any of the training set sites in their environmental conditions that no predictions are made for them.

The predictor variables chosen for the three different cluster versions of Model 2 are shown in Table 2 with their mean ranks across the 100 bootstrap models for each. The average accuracy of these models is shown in Table 3 and the mean values of the selected predictor variables for one of the top models from the 2 Absence: 1 Presence cluster combination is shown in Table 4. Figure 2 shows the mean predicted risk map of LF from the 100 bootstrap models using this same combination of absence and presence clusters. With only one cluster each, LF appeared to be over-predicted whilst with two clusters each LF appeared to be more strongly limited to the training set data points and their immediate surrounding areas (i.e. the disease was possibly under-predicted). The 2 Absence∶1 Presence cluster combination was therefore considered to give the best overall result.

**Table 2:** Mean ranking of the key predictor variables (selected by minimising the AICc) across 100 bootstrap models for each Absence∶Presence cluster combination for Model 2.

	Absence∶Presence clusters					
	1∶1		2∶1		2∶2	
	Variable	Mean rank	Variable	Mean rank	Variable	Mean rank
1	nLST phase 1	7.31	dLST phase1	5.58	dLST min.	7.5
2	Rain phase3	7.56	Rain phase3	8.32	Rain phase2	8.13
3	MIR phase1	7.77	Rain amp3	8.58	nLST amp2	8.67
4	Rain mean	7.78	nLSTamp2	8.78	Rain amp3	8.81
5	Rain amp3	8.07	MIR phase1	8.85	Rain mean	8.95
6	nLST amp2	8.59	Rain mean	9.38	Rain phase3	9.09
7	nLST mean	8.69	nLST variance	9.39	MIR min.	9.11
8	dLST phase3	8.69	nLST mean	9.43	nLST phase1	9.18
9	Rain amp1	9.02	Rain phase1	9.46	dLST amp1	9.4
10	nLST max.	9.39	nLST min.	9.47	NDVI variance	9.42

Key to predictor variable names: MIR = Middle Infrared; dLST = daytime Land Surface Temperature; nLST = nighttime Land Surface Temperature; Rain = Rainfall; NDVI = Normalised Difference Vegetation Index; EVI = Enhanced Vegetation Index. Key to Fourier variable names: Mean = average (observed and predicted); amp1, amp2, amp3 = amplitudes of the annual, bi-annual and tri-annual cycles of changes in the respective variables; phase1, phase2, phase3 = phases (or timing) of the annual, bi-annual and tri-annual cycles of changes in the respective variables; min. = minimum of Fourier fitted value (may therefore be negative); max. = maximum of Fourier fitted value; variance = variance of the raw data.

**Table 3:** Mean accuracy statistics across 100 bootstrap models for each Absence∶Presence cluster combination for Model 2 (see text for definitions) (variables selected by minimising the AICc).

Accuracy	Absence∶Presence clusters		
	1∶1	2∶1	2∶2
Kappa	0.913	0.944	0.982
Sensitivity	97%	97%	99%
Specificity	94%	97%	99%
AUC	0.987	0.991	0.998
AICc	67.5	54.3	30.9

**Table 4:** Example of the mean values of the ten selected variables from one of the 100 bootstrap models for the 2 Absence∶1 Presence cluster situation (Model 2).

	Rain amp3	Rain phase2	nLST min.	Rain min.	Rain amp1	NDVI phase3	MIR phase2	Rain mean	dLST max.	dLST mean	n (sample)
cluster of absence	13.22	4.58	12.82	−7.41	55.22	2.17	4.14	38.16	43.65	37.43	69
cluster of absence	30.33	2.62	17.63	−0.6	96.8	2.01	2.17	143.83	28.65	26.33	30
All absent	18.40	3.99	14.28	−5.34	67.82	2.12	3.54	70.18	39.10	34.07	99
cluster of presence	47.36	7.08	15.58	−14.85	124.84	2.38	2.42	134.53	31.62	27.12	100
All present	47.36	7.08	15.58	−14.85	124.84	2.38	2.42	134.53	31.62	27.12	100
Present & absent	32.96	5.54	14.93	−10.12	96.47	2.25	2.98	102.52	35.34	30.58	199

The variables are given in their order of step-wise selection. See Table 2 for the key to the variable names.

The rainfall variables were disproportionately selected by all cluster combinations in Model 1; each ‘top ten’ list in Table 2 contains four such variables, where the random expectation (5 satellite channels) is only two. At the same time, the vegetation index channels (NDVI and EVI) are under-represented, with only a single one of 20 such variables (10 Fourier variables per channel) chosen across all cluster combinations; the balance of the important predictor variables were thermal ones (either LST or MIR). The relatively high values for the average ranks of even the top variables in all cluster combinations in Table 2, however, reflects the fact that each of the 100 bootstrap samples gave rather different results in terms of the variables selected, and in their order of selection. This is a common feature of relatively small datasets.

Despite the variability in the selected predictor variables, mean model accuracies were very high (Table 3) with, as expected, model accuracy increasing with increasing cluster numbers. The mean values of kappa put all models well within the ‘Excellent’ category of the Landis and Koch [41] scale (where kappa<0.4 is ‘Poor’; 0.4<kappa<0.75 is ‘Good’; and kappa>0.75 is ‘Excellent’).

The mean values of the key predictor variables may differ considerably, or only by rather small amounts (Table 4). Table 4 shows that the mean values for the single clusters of presence points in the model are often intermediate between those of the two absence clusters. This applies to mean rainfall, night-time LST minimum, MIR phase 2 and daytime LST (mean and maximum). In other cases, mean values for the presence points are well outside those for either absence cluster. This applies to rainfall (amp1, amp3, phase1 and minimum) and NDVI phase 3. Concentrating on the important rainfall variables in Table 4 it is possible to suggest that LASV requires high (but not the highest) mean rainfall areas (rain mean), but with very high annual variation of this variable (rain amp1), and with peak rainfall occurring much later in the year (during August rather than during May or March, the months of peak rainfall of the absence clusters in Table 4, rain phase1). The significance of the higher amp3 rainfall value in Table 2 (the first selected variable) is unclear; often such higher harmonics act to modulate the lower frequency – annual or bi-annual – harmonics, and thus adjust the seasonal pattern of rainfall (extending or reducing high rainfall periods, depending on the timing of this tri-annual harmonic).

The predicted risk map (Figure 2) captures most of the presence points in the database (the grey areas in Figure 2 in southern Nigeria and Cameroon are regions where cloud contamination is so continuous that it was not possible to obtain either sufficient cloud-free images or their temporal Fourier derivatives for modelling; these are therefore areas where it is not possible to make predictions of risk). The predicted risk areas in Figure 2 contract towards the coast in the ‘Dahomey gap’ between the western and central forests of Africa (see Introduction) but are still more extensive than the rainfall map and data in Figure 1 suggest. In fact the satellite rainfall image (CMORPH mean, not shown) also indicates a lower mean rainfall area in this region, so that the positive LASV predictions for this area must arise from the values of other key predictor variables. The differences between Figure 1 and Figure 2 in the basin of the River Zaire, towards Central Africa, arise because these areas (though high in rainfall) are environmentally quite distinct from those of the training set area and so the risk map models classify them as ‘No prediction’ areas (coloured grey in Figure 2).

### Model 3

**Figure 3:**
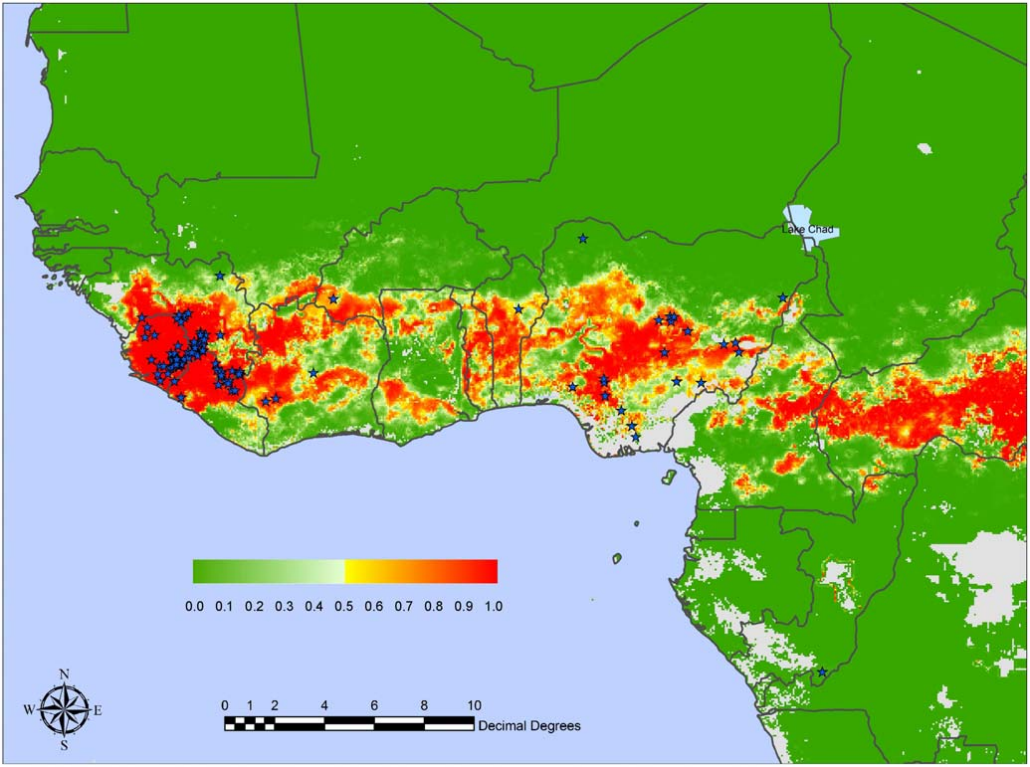
Mean predicted Lassa risk map for West Africa from the Model 3 series with two absence and one presence clusters, with positive localities indicated by stars. Other information as for Figure 2.

Tables 4 and 5 show results analogous to those of Tables 2 and 3 but for Model 3, where the important variables were identified using the combination method of Burnham and Anderson [40]. This method highlights even more the importance of rainfall variables (only 8 out of the 30 variables in Table 5 are not directly rainfall related), with slightly different combinations in each case for the different cluster combinations. Overall model accuracies are still excellent (Table 6) though not quite as good as those for Model 2. Figure 3 shows the mean predicted risk map obtained by using in Model 3 the selected combination of the top 10 variables for the same 100 bootstrap samples that were used in Model 2 to generate Figure 2. Figure 3 is less equivocal about risk areas than is Figure 2 (i.e. there are fewer regions of intermediate probability of LASV risk) for the simple reason that the same 10 variables were used throughout, whereas different combinations of variables were often selected in the Model 2 models, giving more variable results. Figure 3 again captures most of the presence sites within the training set, with rather different predictions for the Dahomey Gap region than those in Figure 2.

**Table 5:** Mean ranking of the key predictor variables across 100 bootstrap models for each Absence∶Presence cluster combination for Model 3.

	Absence∶Presence clusters					
	1∶1		2∶1		2∶2	
	Variable	Mean rank	Variable	Mean rank	Variable	Mean rank
1	Rain phase1	1	Rain amp1	1	EVI variance	1
2	Rain phase2	2	Rain mean	2	Rain variance	2
3	Rain amp1	3	Rain phase1	3	Rain max.	3
4	Rain amp3	4	EVI phase3	4	Rain phase2	4
5	Rain mean	5	Rain max.	5	Rain amp3	5
6	Rain max.	6	Rain phase2	6	NDVI variance	6
7	Rain variance	7	Rain amp3	7	Rain amp1	7
8	Rain amp2	8	dLST phase1	8	dLST mean	8
9	Rain min.	9	Rain min.	9	dLST amp2	9
10	EVI variance	10	EVI variance	10	Rain min.	10

**Table 6:** Mean accuracy statistics across 100 bootstrap models for each Absence∶Presence cluster combination for Model 3 (see text for definitions).

Accuracy	Absence∶Presence clusters		
	1∶1	2∶1	2∶2
Kappa	0.86	0.867	0.917
Sensitivity	93%	94%	96%
Specificity	93%	92%	95%
AUC	0.975	0.98	0.988
AICc	162.8	132	80.4

## Discussion

The question that comes immediately to mind is: why does Lassa fever occur only in West Africa, whereas the range of its vertebrate host extends into East and Southern Africa? This is a recurrent question for other rodent-borne diseases (such as plague and hemorrhagic fevers with renal or pulmonary syndrome; see [42] for a review), which are also much more restricted in their distributions than are their hosts. Our analyses here show quite clearly that Lassa fever requires a particular combination of high (but not the highest) rainfall, and with a particular form of variability and seasonal timing, whereas its hosts can and do occur over regions experiencing a much wider range of rainfall conditions. Temperature appears to be less important in determining LASV distribution, although there are large differences between different areas; for example the annual mean and maxima in high risk areas are 27°C and 32°C respectively, whereas in low risk areas the mean temperature was approx. 38°C. Such high temperatures are known to increase LASV decay [43]. One curious feature of the present results is the seeming unimportance of vegetation variables in the predictor data sets. This lack of importance is not due to their strong correlation with rainfall variables (such a correlation might exclude them in step-wise inclusive variable selection), because Model 3 (using a method that avoids the problems of step-wise methods) independently and quite categorically failed to identify vegetation variables as important in determining LASV distribution. Taken together these results suggest that the survival of the virus outside of the vertebrate host might be a key to determining its distribution, and that this survival depends upon moisture or rainfall conditions above more or less all other environmental variables. This result differs from the conditions favouring other viral transmission; for example, low relative humidity and temperature favour avian influenza [44]. In the case of Lassa, the virus appears to survive better in humid conditions, during the rainy season. Rodents will be more often contaminated during their frequent movements at this season, for mating or dispersing into the surrounding fields [45]. Conversely, viral aerosol stability, seems to be higher when the humidity is lower [43], a condition that obviously occurs more frequently in the dry season. The experiments of Stephenson help to explain the numerous LF cases recorded in hospitals during the late dry season, between January and March in Sierra Leone and Nigeria ([46], Omilabu, pers. com.) but they do not necessarily throw much or any light on the persistence of Lassa fever in the general environment. We suggest that rainfall, within defined limits, is the single most important abiotic determinant of this persistence.

*M. natalensis*, the most important host of LASV, does not occur in the western part of the region, in coastal Guinea and Sierra Leone and west to the 12^th^ meridian. Only *M. erythroleucus* occurs in these regions, and our surveys have always found it to be negative for LASV infections [17]. The low human sero-prevalences recorded in these coastal areas are most likely due to the movement of people from highly endemic zones, or to human-to-human transmission. Towns and villages in these coastal areas, from Guinea to Gabon, have been invaded by the black rat *Rattus rattus*, and the domestic mouse, *Mus musculus*, probably taken there in historical times by Arab and European traders, explorers and colonisers. Absence of *M. natalensis* from coastal areas, for whatever reason (e.g. unsuitable habitats, or competition from other, non-Lassa-reservoir rodents), would explain the absence of Lassa fever in these areas, despite the apparently favourable (for LASV) climatic conditions (although the models suggest that some areas may be too wet for LASV). In Conakry for example, rodent sampling (330 specimens) showed that the most abundant species was *M. musculus* (70%), followed by *R. rattus* (25%) (*unpublished data*).

In East and South Africa, the same reservoir species is present but the virus is replaced by other Lassa-like viruses such as Ippy, Morogoro and Mopeia, found in *M. natalensis* in CAR, Tanzania, Mozambique and Zimbabwe (CRORA database in Pasteur Institute website, http://www.pasteur.fr/recherche/banques/CRORA/, [47],[48],[49]). These different Lassa –like viruses are not known to be pathogenic in humans and are considered ancestral by phylogenetic studies [50]. The scenario of multiple infection with both Lassa-like and Lassa virus is highly unlikely, and so we consider that central and eastern Africa are Lassa free. This is supported by many negative serological studies in Cameroon, in CAR, Congo, Equatorial Guinea and Gabon [24],[25],[26],[27]. However, the situation in south-west Cameroon bordering Nigeria remains problematic because this zone appears to be at high risk according to Figure 2. This is a volcanic area, which could provide a geographic barrier (Mt Cameroon, 4100 m, and the volcano chain up to the Adamaoua plateau). Furthermore, another species of *Mastomys* is suspected to be present in this area, *M. kollmannspergeri*, which is found in Niger, NE Nigeria, N Cameroon, S. Sudan and Chad [51]. In Zakouma National Park in Chad, some specimens were found in a village and in camps, indicating a potential synanthropy of this species [52]. The predictive risk map in Figure 2 identifies the central parts of Cameroon and CAR as risky areas, where it is possible that other Lassa-like viruses could occur, intermediate between Ippy/Mobala and Lassa (Mobala is another Lassa-like virus found in *Praomys sp.*, a closely related species to *Mastomys* spp, in CAR [53].).

According to the risk maps shown here, with the reservations noted above, the LF risk area covers approximately 80% of the area of each of Sierra Leone and Liberia, 50% of Guinea, 40% of Nigeria, 30% of each of Côte d'Ivoire, Togo and Benin and 10% of Ghana. Such maps help public health policies and research, in targeting disease control and studies in potentially infected areas.

## Supporting Information

Alternative Language Abstract S1 Translation of the abstract into French by Elisabeth Fichet-Calvet
(0.02 MB DOC)
