# Supplementary material for: Risk Maps of Lassa Fever in West Africa
Source: PLoS Negl Trop Dis. 2009 Mar 3;3(3):e388. doi: 10.1371/journal.pntd.0000388 (PMC2644764; doi:10.1371/journal.pntd.0000388)
Supplement: Alternative Language Abstract S1 — Translation of the abstract into French by Elisabeth Fichet-Calvet (0.02 MB DOC) [file pntd.0000388.s001.doc]

# Supporting information:

# Translation of the abstract into French by author EFC

# Contexte

# La fièvre de Lassa est une fièvre hémorragique virale, qui affecterait 2 à 3 millions de personnes en Afrique de l’ouest, causant la mort de 5000 à 10000 d’entre elles. Elle est due à un arenavirus dont le réservoir et vecteur est le rat à mamelles multiples, *Mastomys natalensis*, qui vit dans les maisons et les champs environnants. Dans le but de cibler des campagnes d’information et de lutte contre cette maladie, nous proposons une analyse épidémiologique spatiale afin de dresser une carte de risque, basée sur des variables environnementales à l’échelle du continent africain. Une large revue de la littérature a permis d’acquérir les points positifs où la maladie a été sûrement identifiée. Ces points sont alors référencés selon leur latitude et longitude. Ensuite différentes variables environnementales comme la pluviométrie, la température, la végétation et l’altitude sont acquises sur toute la zone sahélienne et tropicale entre le Sénégal et le Congo. Les données pluviométriques proviennent de stations météorologiques pour la période 1951-1989, et du satellite Terra (NASA) pour la période 2000-2005. Température et végétation (proche et moyen infra-rouge) proviennent également du satellite Terra, alors que l’altitude provient de la base GTOPO30. Toutes les données satellitaires sont transformées selon la technique temporelle de Fourier pour générer des images de moyennes, amplitudes et phases, lesquelles seront utilisées comme variables prédictrices dans les modèles.

# Méthodologie

# Trois modèles (cartes) sont alors proposés et dépendent du traitement statistique sous-jacent. Le modèle 1 est issu d’une simple superposition entre données épidémiologiques et pluviométriques (moyenne annuelle) alors que les deux autres utilisent une analyse discriminante non linéaire, dans le cadre d’un bootstrap (100 échantillons différents issus du jeu de données). Le modèle 2 inclut une sélection de 10 variables obtenues par inclusion au pas à pas ascendante, tandis que le modèle 3 inclut une sélection des 10 meilleures variables obtenues à partir de combinaisons de 10 variables tirées au hasard. Le critère d’Akaike moyen (corrigé pour la taille de l’échantillon et le nombre de variables incluses) le plus bas, est utilisé pour évaluer la meilleure corrélation entre données épidémiologiques et environnementales. Trois autres critères, indice de Kappa, sensitivité et spécificité, servent aussi à évaluer la pertinence de chaque modèle. Enfin, trois combinaisons de grappes, une ou deux absence pour une ou deux présence ont été utilisées dans chacun des modèles 2 et 3, la grappe deux absence pour une présence donnant apparemment le meilleur résultat.

Principaux résultats/Conclusion

Le modèle 1 montre que les cas de fièvre de Lassa sont situés dans les régions recevant entre 1500 et 3000 mm d’eau par an. Les modèles 2 et 3 montrent que les variables liées à la pluviométrie sont fortement sélectionnées, puis dans une moindre mesure celles liées à la température, alors que la végétation et l’altitude semblent sans importance. Les modèles 2 et 3 produisent des indices de Kappa excellents de l’ordre de 0,91 et 0,86 respectivement. La sensitivité et la spécificité de tels modèles atteignent aussi des valeurs élevées, égales ou supérieures à 92%. La carte issue du modèle 3 est légèrement différente de celle du modèle2, car les zones à moyen risque sont inexistantes et des zones à haut risque apparaissent dans des zones sahéliennes comme le nord de la Côte d’Ivoire et le sud du Burkina Faso. En considérant quelques restrictions comme l’absence du réservoir dans les zones côtières d’Afrique de l’ouest, ainsi que la probable absence du virus Lassa en Afrique Centrale, ces nouvelles cartes de risque permettent de suggérer la zone Lassa couvrant 80% de la Sierra Leone, 50% de la Guinée et du Liberia, 40% du Nigeria, 30% de la Côte d’Ivoire, du Togo et du Benin et 10% du Ghana.
